# Supplementary material for: The feasibility of an objective measure of the parent-child relationship in health visiting practice: assessment of the Maternal Postnatal Attachment Scale
Source: Wellcome Open Res. 2022 Nov 7;7:88. Originally published 2022 Mar 11. [Version 2] doi: 10.12688/wellcomeopenres.17552.2 (PMC9664022; doi:10.12688/wellcomeopenres.17552.2)
Supplement: Peer review history

# Peer review: Reviewer response for version 2

**DOI:** 10.21956/wellcomeopenres.20368.r53197

**Published:** 2022-11-14

**Authors:** Nicola A. Savory

**Affiliations:** 1 Maternity Unit, University Hospital of Wales, Heath Park, Cardiff and Vale University Health Board, Cardiff, UK

Thank you for the changes which make this article easier for the reader to understand. 

 I have no further comments to make.

Is the work clearly and accurately presented and does it cite the current literature?

Yes

If applicable, is the statistical analysis and its interpretation appropriate?

I cannot comment. A qualified statistician is required.

Are all the source data underlying the results available to ensure full reproducibility?

Partly

Is the study design appropriate and is the work technically sound?

Yes

Are the conclusions drawn adequately supported by the results?

Yes

Are sufficient details of methods and analysis provided to allow replication by others?

Partly

Reviewer Expertise:

My areas of research include, maternity care throughout the perinatal period. Within this I have reviewed service provision, maternal and midwives experiences and mental health. Methods include multiple method research, questionnaires, interviews and focus groups, use of routine data.

I confirm that I have read this submission and believe that I have an appropriate level of expertise to confirm that it is of an acceptable scientific standard.

# Peer review: Reviewer response for version 2

**DOI:** 10.21956/wellcomeopenres.20368.r53196

**Published:** 2022-11-07

**Authors:** Eleonora Bielawska-Batorowicz

**Affiliations:** 1 Department of Clinical Psychology and Psychopathology, Faculty of Educational Sciences, Institute of Psychology, University of Lodz, Lodz, Poland

I accept the amended version of the paper

Is the work clearly and accurately presented and does it cite the current literature?

Yes

If applicable, is the statistical analysis and its interpretation appropriate?

Yes

Are all the source data underlying the results available to ensure full reproducibility?

Yes

Is the study design appropriate and is the work technically sound?

Yes

Are the conclusions drawn adequately supported by the results?

Yes

Are sufficient details of methods and analysis provided to allow replication by others?

Yes

Reviewer Expertise:

reproductive psychology, parental prenatal and postnatal attachment, reproductive mental health, psychology of menopause

I confirm that I have read this submission and believe that I have an appropriate level of expertise to confirm that it is of an acceptable scientific standard.

# Peer review: Reviewer response for version 1

**DOI:** 10.21956/wellcomeopenres.19409.r51297

**Published:** 2022-07-12

**Authors:** Nicola A. Savory

**Affiliations:** 1 Maternity Unit, University Hospital of Wales, Heath Park, Cardiff and Vale University Health Board, Cardiff, UK

**Reviewers comments**

 Thank you for the opportunity to review this manuscript which seeks to look at the feasibility/acceptability, validity, and reliability of a screening tool to assess parent-infant relationships. The gap in evidence for a new tool for maternal-infant attachment is clearly laid out. A description of the role of the health visitor in the UK is provided for international readers. There is mention of the clinical relevance and need for a validated screening tool.

 This is a well-written paper. I only have two comments.

1. In the introduction, there is mention of prevalence rates for insecure attachment or attachment disorders. There is such a  significant difference in these prevalence rates 1.4% and 35%. Could a sentence be added to explain the possible reasons for this difference. Was it related to reporting methods, were they well reported papers?
2. The other comment relates to the ease of reading the manuscript. I have read the methods/results section a couple of times and I am still trying to distinguish if there are two different groups – those eligible to participate and BiBBS participants. Or are the BiBBS participants a sub-group of the total number of participants? It is not clear in the Abstract which only reports on 563 women in BiBBS group. Could this be made clearer for the reader throughout the paper?

Is the work clearly and accurately presented and does it cite the current literature?

Yes

If applicable, is the statistical analysis and its interpretation appropriate?

I cannot comment. A qualified statistician is required.

Are all the source data underlying the results available to ensure full reproducibility?

Partly

Is the study design appropriate and is the work technically sound?

Yes

Are the conclusions drawn adequately supported by the results?

Yes

Are sufficient details of methods and analysis provided to allow replication by others?

Partly

Reviewer Expertise:

My areas of research include, maternity care throughout the perinatal period. Within this I have reviewed service provision, maternal and midwives experiences and mental health. Methods include multiple method research, questionnaires, interviews and focus groups, use of routine data.

I confirm that I have read this submission and believe that I have an appropriate level of expertise to confirm that it is of an acceptable scientific standard, however I have significant reservations, as outlined above.

# Peer review: Reviewer response for version 1

**DOI:** 10.21956/wellcomeopenres.19409.r49695

**Published:** 2022-04-20

**Authors:** Eleonora Bielawska-Batorowicz

**Affiliations:** 1 Department of Clinical Psychology and Psychopathology, Faculty of Educational Sciences, Institute of Psychology, University of Lodz, Lodz, Poland

The measurement of postnatal parental attachment requires a reliable tool and even more so in the context of screening in the health visiting setting. The manuscript describes the process of validation of one of the measures of postnatal maternal attachment (Maternal Postnatal Attachment Scale by Condon & Corkindale) in the sample of women from a disadvantaged and ethnically diverse community. The aims and rationale of the study are clearly stated and the methodology is properly described. It is interesting that the analyses were conducted in two ways:

1. by assessing the feasibility and acceptability of implementation of MPAS in a routine health visiting practice and,  
2. by assessing the validity and reliability of MPAS. The results of both analyses are properly presented and illustrated by figures, tables, and relevant statistical data.

Although I do not consider myself to be an expert in statistical analyses, the way the validity and reliability of MPAS have been evaluated seems to be properly designed and conducted. Therefore the findings are convincing and conclusions appropriate for the data presented in the text. The strength and limitations were clearly discussed therefore the final conclusions on the feasibility of the usage of MPAS in health visiting practice (especially in the studied community) and its validity are appropriate for the results of the study. It is interesting that the Authors follow their findings by engaging in the construction and validation of another tool aimed to assess the parent-child relationship in the context of routine postnatal health care.

Is the work clearly and accurately presented and does it cite the current literature?

Yes

If applicable, is the statistical analysis and its interpretation appropriate?

Yes

Are all the source data underlying the results available to ensure full reproducibility?

Yes

Is the study design appropriate and is the work technically sound?

Yes

Are the conclusions drawn adequately supported by the results?

Yes

Are sufficient details of methods and analysis provided to allow replication by others?

Yes

Reviewer Expertise:

reproductive psychology, parental prenatal and postnatal attachment, reproductive mental health, psychology of menopause

I confirm that I have read this submission and believe that I have an appropriate level of expertise to confirm that it is of an acceptable scientific standard.
